# Comments on: Validating the Heat Stress Indices for Using In Heavy Work Activities in Hot and Dry Climates

**Published:** 2016-09-21

**Authors:** Erfan Ayubi, , Kamyar Mansori

**Affiliations:** ^a^ Department of Epidemiology, School of Public Health, Shahid Beheshti University of Medical Sciences, Tehran, Iran; ^b^ Department of Epidemiology & Biostatistics, School of Public Health, Tehran University of Medical Sciences, Tehran, Iran; ^c^ Social Determinants of Health Research Center, Kurdistan University of Medical Sciences, Sanandaj, Iran.; ^d^ Department of Epidemiology, School of Public Health, Iran University of Medical Sciences, Tehran, Iran

## Dear Editor-in-Chief

We read with great interest the paper entitled validating the Heat Stress Indices for Using in Heavy Work Activities in Hot and Dry Climates published online in Journal of Research in Health Sciences in Jun 2016^1^. The authors aimed to assess the precision and validity of some heat stress indices and select the optimum index for using in heavy work activities in hot and dry climates. According to their paper, there is a close correlation between wet-bulb globe temperature (WBGT) with predicted heat strain (*r*=0.93) and heat stress index (*r*=0.93).

Although the statistical method is correct and data are interesting but some methodological and statistical issues should be considered. The authors used Pearson correlation coefficient as indicator of validity by comparing WBGT with the heat stress indices. Considering their conclusion, WBGT can be introduced as the most valid empirical index of heat stress in the brick workshops. Such a conclusion has nothing to do with validity analysis when WBGT is compared with predicted heat strain and heat stress index because these indexes have different scales.

**Figure 1:**
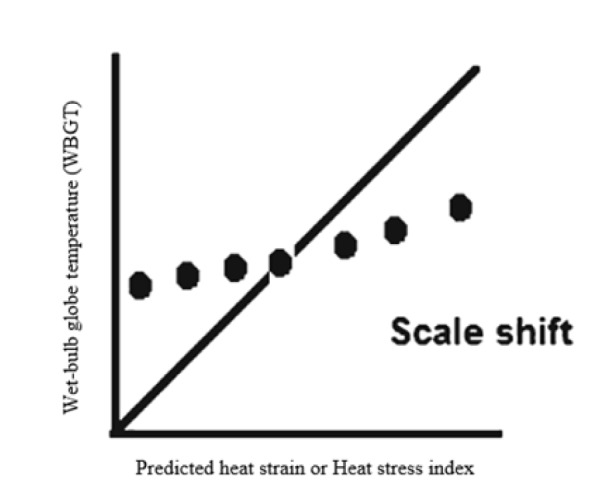
Pearson correlation coefficient show perfect correlation but it is not necessarily a good measure of validity

Using the Pearson correlation coefficient may be misleading because it is insensitive to systematic difference of WBGT, predicted heat strain and heat stress index in measuring heat stress. On the other hand, compared to WBGT, predicted heat strain and heat stress index may tend to measure higher value of heat stress at lower level or vice versa. In manner of biostatistics the probable explanation for observed *r*=0.93 is that the points falls in a perfect line but the slope of the line is different from 1.0 (Figure 1)^2^.

Moreover, instead of Pearson correlation coefficient that has its own limitations, there are efficient and advanced methods such as explanatory factor analysis (EFA) and confirmatory factor analysis to evaluate validity^3^. We suggest that the authors should consider reanalyzing their data with the hypothesis of does WBGT contributed to other heat stress through common factors. First, the authors can run an EFA on WBGT and other heat stress indices and second, the extracted factors be tested by CFA in an independent sample.

As a take home message, for validity analysis, efficient statistical methods as well as correct interpretation should be applied.

Erfan Ayubi (MSc)^a,b^*

^a^ *Department of Epidemiology, School of Public Health, Shahid Beheshti University of Medical Sciences, Tehran, Iran*

^b^ *Department of Epidemiology & Biostatistics, School of Public Health, Tehran University of Medical Sciences, Tehran, Iran*

^c^ *Social Determinants of Health Research Center, Kurdistan University of Medical Sciences, Sanandaj, Iran.*

^d^ *Department of Epidemiology, School of Public Health, Iran University of Medical Sciences, Tehran, Iran*

*****Correspondence to:***** *Kamyar Mansori (MSc)*

**E-mail:** kamyarmansori@yahoo.com

## Reply

Thank you for the opportunity to respond to the Letter to the Editor article entitled “Validating the Heat Stress Indices for Using in Heavy Work Activities in Hot and Dry Climates: Methodological issues of validity” published online in Journal of Research in Health Sciences. We also appreciate the scientific reader’s efforts who carefully reviewed the article and the valuable suggestions offered.

The readers mentioned that some methodological and statistical issues should be considered in our article to prove that WBGT can be introduced as the most valid empirical index of heat stress in the brick workshops and indicated that such a conclusion about the most valid empirical index has nothing to do with validity analysis when WBGT compared with predicted heat strain and heat stress index because these indexes have different scales. They suggested that we should consider reanalyzing their data with the hypothesis of does WBGT contributed to other heat stress through common factors.

In response, we do not determine the validity of heat stress index based on its correlation coefficient with other heat stress indices. In our study, the validity of indices has been evaluated according to their correlation with physiological parameters, and also considering some other factors like their ease of measurement, and precision based on changes in environmental parameters.

Among Wet-bulb globe temperature (WBGT), predicted heat strain and heat stress index which had the highest correlation with physiological parameters, we offered WBGT index, because it is the most applicable index for assessing heat stress in workplaces and it is approved by ISO, and it has some positive features such as ease of measurement and calculation; moreover, with respect to some limitation in application of HSI.

***Corresponding author:*** *Farideh Golbabaei (PhD)*

**Email:** fgolbabaei@sina.tums.ac.irGolbabaei

## References

[1] Hajizadeh R, Golbabaei F, Farhang Dehghan S, Beheshti MH, Jafari SM, Taheri F (2016). Validating the heat stress indices for using in heavy work activities in hot and dry climates. J Res Health Sci.

[2] Szklo M, Nieto J. Epidemiology: beyond the basics. 3^rd^ ed. Burlington: Jones & Bartlett Publishers; 2012.

[3] Munro BH. Statistical methods for health care research: Lippincott Williams & Wilkins; 2005.

